# Fully automated segmentation and volumetric measurement of ocular adnexal lymphoma by deep learning-based self-configuring nnU-net on multi-sequence MRI: a multi-center study

**DOI:** 10.1007/s00234-024-03429-5

**Published:** 2024-07-17

**Authors:** Guorong Wang, Bingbing Yang, Xiaoxia Qu, Jian Guo, Yongheng Luo, Xiaoquan Xu, Feiyun Wu, Xiaoxue Fan, Yang Hou, Song Tian, Sicong Huang, Junfang Xian

**Affiliations:** 1 Department of Radiology, Beijing Tongren Hospital, Capital Medical University, No.1 DongJiaoMinXiang Street, DongCheng District, Beijing, 100730 China; 2 Department of Radiology, The Second Xiangya Hospital, Central South University, Changsha, China; 3 Department of Radiology, The First Affiliated Hospital of Nanjing Medical University, Nanjing, China; 4 Department of Radiology, Shengjing Hospital of China Medical University, Shenyang, China; 5 Philips Healthcare, Beijing, China

**Keywords:** Ocular adnexal lymphoma, Deep learning, Magnetic resonance imaging

## Abstract

**Purpose:**

To evaluate nnU-net’s performance in automatically segmenting and volumetrically measuring ocular adnexal lymphoma (OAL) on multi-sequence MRI.

**Methods:**

We collected T1-weighted (T1), T2-weighted and T1-weighted contrast-enhanced images with/without fat saturation (T2_FS/T2_nFS, T1c_FS/T1c_nFS) of OAL from four institutions. Two radiologists manually annotated lesions as the ground truth using ITK-SNAP. A deep learning framework, nnU-net, was developed and trained using two models. Model 1 was trained on T1, T2, and T1c, while Model 2 was trained exclusively on T1 and T2. A 5-fold cross-validation was utilized in the training process. Segmentation performance was evaluated using the Dice similarity coefficient (DSC), sensitivity, and positive prediction value (PPV). Volumetric assessment was performed using Bland-Altman plots and Lin’s concordance correlation coefficient (CCC).

**Results:**

A total of 147 patients from one center were selected as training set and 33 patients from three centers were regarded as test set. For both Model 1 and 2, nnU-net demonstrated outstanding segmentation performance on T2_FS with DSC of 0.80–0.82, PPV of 84.5–86.1%, and sensitivity of 77.6–81.2%, respectively. Model 2 failed to detect 19 cases of T1c, whereas the DSC, PPV, and sensitivity for T1_nFS were 0.59, 91.2%, and 51.4%, respectively. Bland–Altman plots revealed minor tumor volume differences with 0.22–1.24 cm^3^ between nnU-net prediction and ground truth on T2_FS. The CCC were 0.96 and 0.93 in Model 1 and 2 for T2_FS images, respectively.

**Conclusion:**

The nnU-net offered excellent performance in automated segmentation and volumetric assessment in MRI of OAL, particularly on T2_FS images.

**Supplementary Information:**

The online version contains supplementary material available at 10.1007/s00234-024-03429-5.

## Introduction

Ocular adnexal lymphoma (OAL) is a common orbital malignant tumor in adults and accounts for 11–55% of orbital malignancies [1–3]. Over the past few decades, there has been a marked rise in the incidence of OAL, adversely affecting the quality of life for affected individuals [3–6]. Presently, radiotherapy is commended as an important treatment strategy for OAL in clinics, as it can easily pinpoint and shrink the tumor [7–9]. Determining the tumor boundaries and volume has essential benefits for radiation therapy planning and treatment efficiency evaluation [10, 11].

Prior studies have demonstrated that magnetic resonance imaging (MRI), which offers both quantitative and qualitative information for diagnosis and guided treatment, represents the optimal non-invasive examination approach for orbital tumors [12–14]. MRI plays a significant role in delineating tumor boundaries, not only for initial diagnosis but also for monitoring treatment progress and making necessary adjustments based on the tumor’s characteristics. However, in typical clinical settings, radiologists often rely on subjective assessments of tumor size on MRI images, lacking objective tumor volume data. This is primarily due to the laborious and time-consuming nature of manual segmentation, which may introduce inter-observer and intra-observer errors. Thus, the precise and automated recognition of tumor contours and the measurement of tumor volume on MRI images may serve as a pivotal factor in optimizing the daily clinical workflow for patients with OAL.

Recent research suggests that deep neural networks can process multi-modal data [15, 16]. For MRI analysis, convolutional neural networks have been explored for multi-sequence modeling and have proven effective [17]. However, the complexity of deep learning involves configuring numerous parameters, posing a challenge for non-experts in model training. To address this, recent years have seen the development of fully automated deep learning frameworks and pipelines for doctors and radiologists, such as nnU-net, enabling training without extensive coding [18].

This study aimed to develop a multi-sequence deep neural network, utilizing a fully automated self-configuring training framework, nnU-net, for segmenting OAL and measuring its volume on a multi-sequence MRI dataset.

## Methods

### Study design and participating datasets

This retrospective study obtained approval from the hospital’s institutional review board (No. TREC2023-KY107). Given its inherently retrospective nature, the requirement for written informed consent was waived.

MRI images of patients with ocular adnexal lymphoma (OAL) were retrospectively collected between January 2015 and March 2022 from our institution. Inclusion criteria consisted of (1) histopathologically confirmed diagnosis of OAL; (2) a comprehensive MRI image dataset, including axial T1- and T2-weighted images (T1, T2) and T1-weighted contrast-enhanced (T1c) sequences; (3) presence of identical OAL lesions on MRI images. Exclusion criteria included: (1) severe motion artifacts impeding accurate interpretation; (2) small lesions occupying fewer than consecutive two slices; and (3) lesions lacking a clear pathological diagnosis. All images were anonymized and exported in Digital Imaging and Communication in Medicine (DICOM) format.

**Fig. 1:**
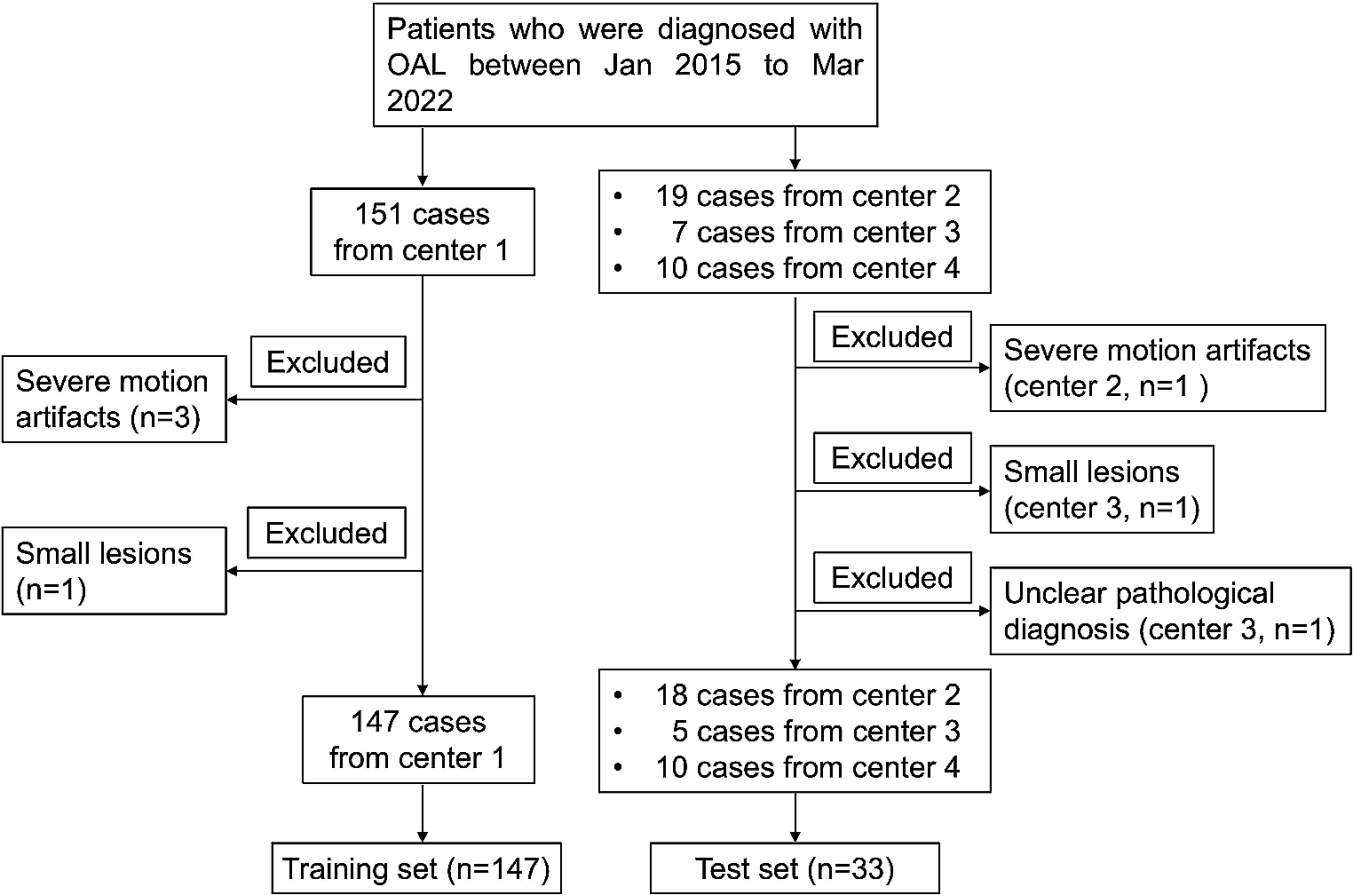
Flowchart of the patient selection

Based on the aforementioned inclusion and exclusion criteria, a total of 147 patients from Center 1 were enrolled in the training set. Furthermore, thirty-three OAL patients from three separate institutions (Centers 2–4) meeting the aforementioned criteria during the same period were selected as the external test set. A data-sharing protocol was established and signed among the participating centers. The study workflow is illustrated in Fig. 1.

### MRI acquisition

The training dataset (*n* = 147) was acquired using 1.5T (Philips Ingenia, *n* = 2) and 3T scanners [(1) Philips Ingenia, *n* = 83; (2) GE Discovery MR750, *n* = 50; (3) Siemens Magnetom Prisma, *n* = 12)] from various MRI manufacturers. The test dataset (*n* = 33) was obtained exclusively from 3T scanners: (1) Center 2 (*n* = 18): GE Discovery MR750 (*n* = 5), Siemens Somatom Skyra (*n* = 8), and Siemens Magnetom Verio (*n* = 5); (2) Center 3 (*n* = 5): Philips Ingenia; (3) Center 4 (*n* = 10): United Imaging uMR790 (*n* = 2), Philips Achieva (*n* = 1), and Siemens Somatom Skyra (*n* = 7). Due to variations in scanning protocols across different centers, T2 and T1c images were presented in two forms: with and without fat saturation (FS/nFS). However, each patient had only one form of T2 or T1c images. T1c images were acquired by intravenous injection of a gadolinium-based contrast agent at a dose of 0.1 mmol/L. Detailed information on the MRI scanners and sequences is provided in Table 1, with corresponding protocols outlined in Supplementary Table S1.

**Table 1:** MRI scanners and sequences of the training set and test set

	MRI scanners	MRI sequences				
		T1	T2_FS	T2_nFS	T1c_FS	T1c_nFS
Training set (*n* = 147)		147	83	64	147	0
Center 1 (*n* = 147)	Philips Ingenia, 3.0T	83	80	3	83	0
	GE Discovery MR750, 3.0T	50	3	47	50	0
	Siemens Magnetom Prisma, 3.0T	12	0	12	12	0
	Philips Ingenia, 1.5T	2	0	2	2	0
Test set (*n* = 33)		33	30	3	19	14
Center 2 (*n* = 18)	GE MR750, 3.0T	5	5	0	5	0
	Siemens Somatom Skyra, 3.0T	8	8	0	0	8
	Siemens Magnetom Verio, 3.0T	5	5	0	0	5
Center 3 (*n* = 5)	Philips Ingenia, 3.0T	5	2	3	4	1
Center 4 (*n* = 10)	United Imaging uMR790, 3.0T	2	2	0	2	0
	Philips Achieva, 3.0T	1	1	0	1	0
	Siemens Somatom Skyra, 3.0T	7	7	0	7	0

Data are presented as the number of cases

### Manual segmentation

All segmentations of OAL lesions were initially conducted by a junior radiologist (Radiologist 1) with four years of experience in head and neck imaging. The regions of interest (ROIs) along the boundaries of OAL on multi-sequence MRI images (T1, T2_FS or T2_nFS, T1c_FS or T1c_nFS) were outlined slice by slice using ITK-SNAP (version 4.0.0, http://www.itksnap.org/pmwiki/pmwiki.php). Subsequently, a senior radiologist (Radiologist 2) with eight years of experience in head and neck imaging reviewed and validated the initial annotations. Any discrepancies were resolved through discussions or consultation with another experienced expert to achieve consensus. The manually segmented results that obtained consensus were deemed to be the ground truth. Furthermore, to assess intra-observer consistency, we randomly selected 30 cases for re-segmentation by Radiologist 1 after one month.

### Data processing

All scans were automatically resampled to a consistent voxel spacing of 0.31 × 0.31 × 3.00 mm^3^ (the median spacing across scans). Subsequently, a Z-score normalization was applied to standardize the distribution of intensities. We employed a patch-based training strategy adapted from the NIFTYNET framework [19]. Given a median image dimension of 16 × 506 × 511 voxels, the patch size was automatically set to 12 × 384 × 384 voxels.

Online spatial data augmentation encompassed random flipping, rotation, and affine transformations. Intensity augmentations included Gaussian blurring, contrast adjustment, low-resolution simulation, and gamma transformation with default parameters. The calculation of tumor volume involved multiplying the pixel by the product of voxel spacing.

### Model development

In the development of MRI segmentation models for OAL, we employed the nn-UNet V2 pipeline (version 2.1) [18]. Considering patients in clinical settings may lack contrast-enhanced MRI images due to contrast agent allergies or other reasons, we developed and trained two distinct models to broaden the algorithm’s applicability. The first, labeled “Model 1,” was trained on T1, T2, and T1c images, while the second, labeled “Model 2,” exclusively used T1 and T2 images. The input channel for both models was set to 1. Both models employed a five-fold cross-validation approach during the training process [20–23]. Specifically, the training dataset was split into 5 nonoverlapping folds, with each fold used for validation while the remaining four folds were utilized for training. This resulted in the creation of five sub-models, where each fold was utilized once for testing and four times for training. Importantly, the final selection of the model for each variation was determined based on its performance in internal validation across each fold. At the inference stage, the OAL segmentation was achieved by averaging the predictions from the 5 sub-models using the nnUNetv2_ensemble module (Supplementary Fig. S1).

**Fig. 2:**
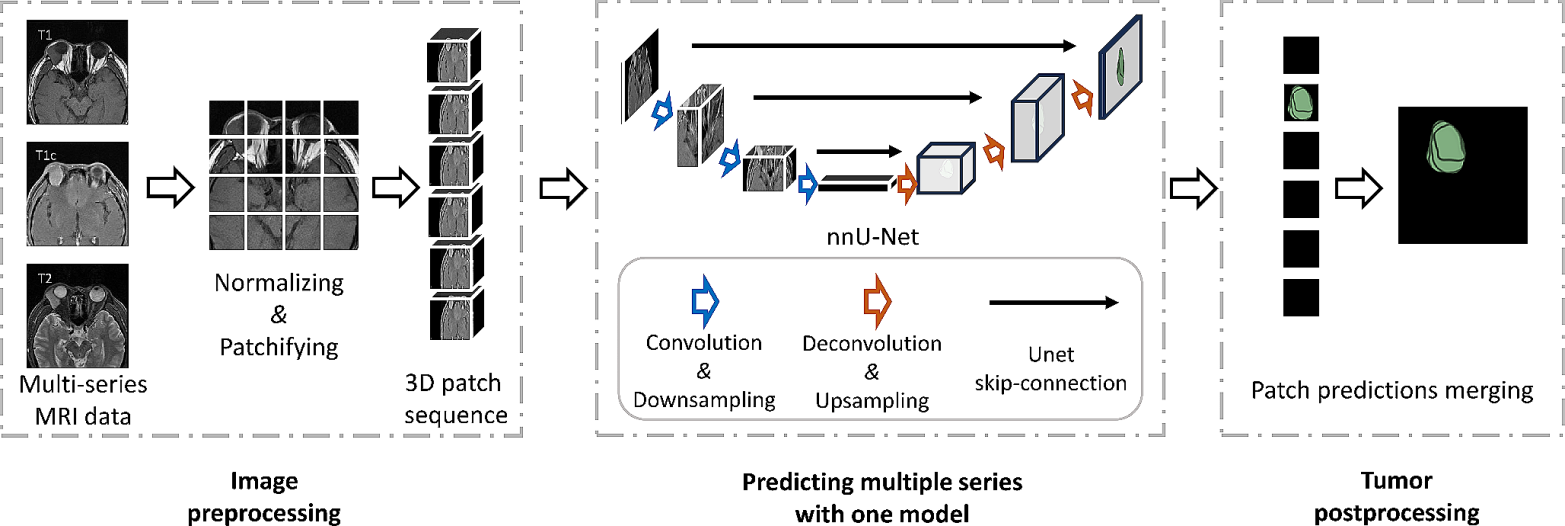
The processing workflow of nnU-net for OAL segmentation and volumetric assessment

The training configuration employed the “3d_fullres” mode with the default nnU-Net V2 sampling strategy, randomly selecting 250 patches containing the OAL region per epoch. Each model underwent 1000 epochs of training with a batch size of 2. The training process was executed on two GPUs, each equipped with 24 GB of graphics memory, using PyTorch (version 2.0.0, https://pytorch.org/get-started/locally/) and CUDA (version 11.7, https://developer.nvidia.com/cuda-11-7-0-download-archive). The workflow of data processing is shown in Fig. 2.

### Statistical analysis

Statistical analysis was conducted by Python (version 3.9) and SPSS (version 20.0, SPSS Inc., Chicago, USA). To assess the normality of the data distribution, the Shapiro-Wilk test was employed. Data not conforming to a normal distribution were expressed as the median and quartile ranges, or else were summarized as mean ± standard deviation (SD). The t-test and chi-square test were used to compare the continuous and categorical variables, respectively. The performance of nnU-net was evaluated by the Dice similarity coefficient (DSC, ranging from 0 to 1), sensitivity, and positive prediction value (PPV). Bland–Altman plots were generated using the volumes of the ground truth and predicted tumor, and Lin’s concordance correlation coefficient (CCC) was employed to evaluate the consistency between them. The consistency between nnU-net prediction and manual segmentation was interpreted as almost perfect (> 0.99), substantial (0.95 ~ 0.99), moderate (0.90 ~ 0.95), and poor (< 0.90) [24]. The intra-observer agreement and the consistency of tumor volume obtained through manual segmentation across the three MRI sequences were evaluated using the intraclass correlation coefficient (ICC) with a two-way mixed absolute agreement model. The interpretation of ICC values was as follows: poor (ICC < 0.5), moderate (0.5 ≤ ICC < 0.75), good (0.75 ≤ ICC < 0.9), and excellent (ICC ≥ 0.9) [25]. A *p* value less than 0.05 is regarded as a statistical difference.

## Results

### Patient characteristics

The characteristics of the patients included in this study are presented in Table 2. There were no significant differences in terms of sex (*p* = 0.327) or the side of the lesion location (*p* = 0.496) between the training set and test set. Patients in the test set were younger than those in the training set (*p* = 0.030), and a statistically significant difference in the distribution of tumor volume between these two datasets was observed (*p* = 0.022). The intra-observer agreement demonstrated great consistency with an ICC range of 0.889 to 0.956, indicating the robust stability of ROI delineation. Tumor volume by manual annotation among the three MRI sequences showed excellent consistency, with an ICC of 0.945.

**Table 2:** Patient characteristics of the training set and test set

	Training set (*n* = 147)	Test set (*n* = 33)	*p*
Age	61.3 ± 12.2	56.1 ± 12.6	0.030
Sex			0.327
Men	60.5 (89/147)	69.7 (23/33)	
Women	39.5 (58/147)	30.3 (10/33)	
Tumor volume (cm^3^)			0.022
<10	79.6 (117/147)	60.6 (20/33)	
10–20	17.7 (26/147)	30.3 (10/33)	
20–30	1.4 (2/147)	9.1 (3/33)	
> 30	1.4 (2/147)	0 (0/33)	
Side			0.496
Left	47.6 (70/147)	36.4 (12/33)	
Right	34.0 (50/147)	42.4 (14/33)	
Bilateral	18.4 (27/147)	21.2 (7/33)	

Data are presented as mean ± SD or percentages, with numbers are used calculating percentages in parentheses

### Evaluation of performance of training set

Using 5-fold cross validation, nnU-net achieved a DSC in the range of 0.78–0.87 considering all MRI sequences in Model 1. The volumetric difference between nnU-net prediction and the ground truth was 0.02–1.28 cm^3^, with the CCC of the five sub-models ranging from 0.92 to 0.95. For Model 2, nnU-net obtained DSC ranging from 0.75 to 0.86, with a volumetric variance of 0.08–1.71 cm^3^. The CCC was 0.84–0.89 for all of the sub-models in Model 2 (Supplementary Fig. S2, Table S2-S3).

### Performance of Model 1 for automated segmentation

**Fig. 3:**
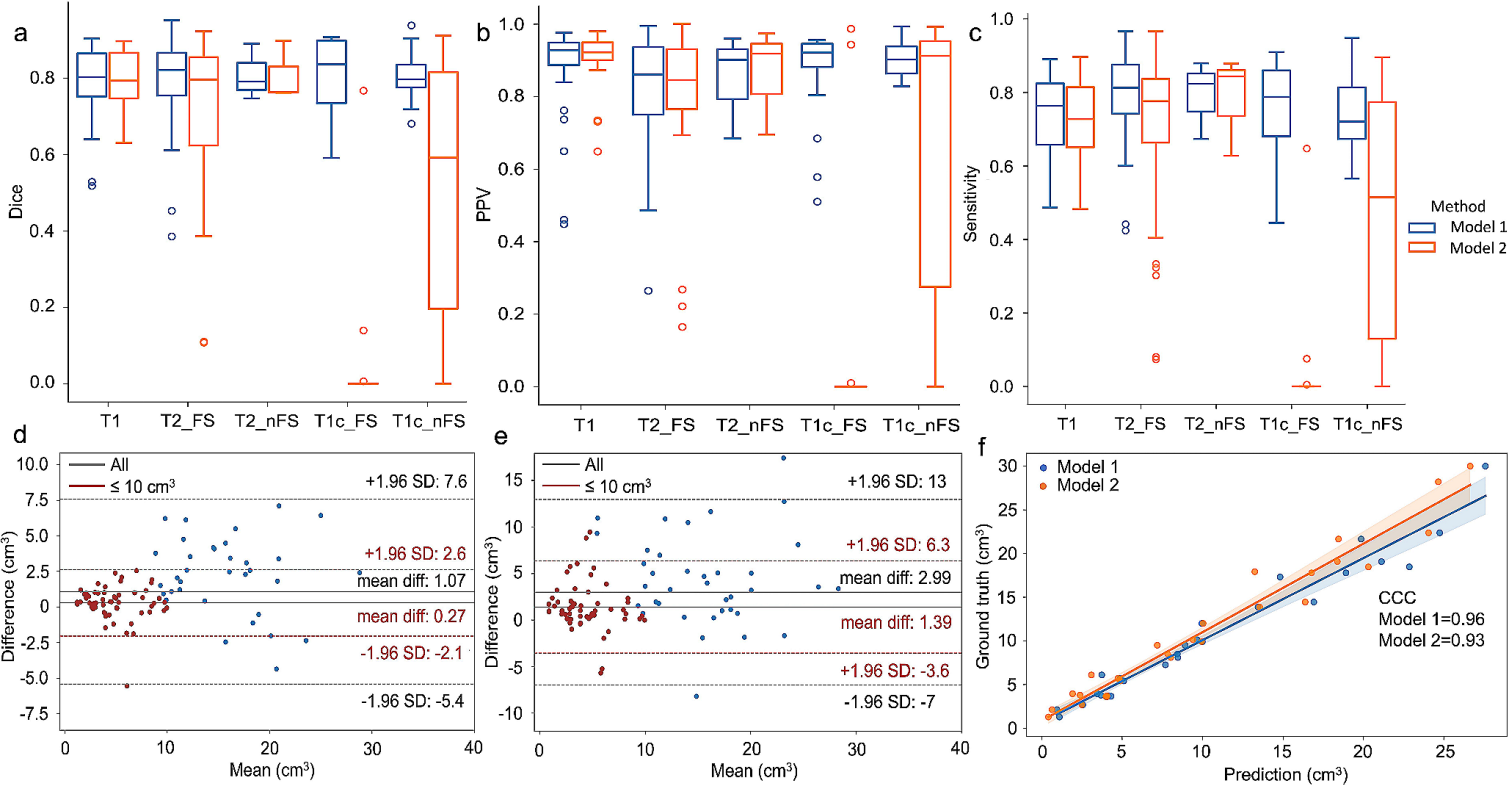
The segmentation performance of nnU-net for OAL. The values of Dice (**a**), PPV (**b**), and sensitivity (**c**) of multi-sequence MRI images in Model 1 and Model 2. Outliers are represented as circles. Bland-Altman plots of volumetric comparisons between nnU-net predictions and the ground truth defined by radiologists in Model 1 (**d**) and Model 2 (**e**). The mean differences between nnU-net and manual segmentation are 1.07 cm^3^ and 2.99 cm^3^ for all lesions in Model 1 and Model 2, respectively. In the cases of smaller lesions with a volume less than 10 cm^3^, the mean differences between predicted and ground truth are 0.27 cm^3^ and 1.39 cm^3^ in Model 1 and Model 2, respectively. The concordance correlation coefficients (CCCs) of tumor volume between the prediction by nnU-net and the manual segmentation in Model 1 and Model 2 on T2_FS images (**f**) are 0.96 and 0.93, respectively

**Fig. 4:**
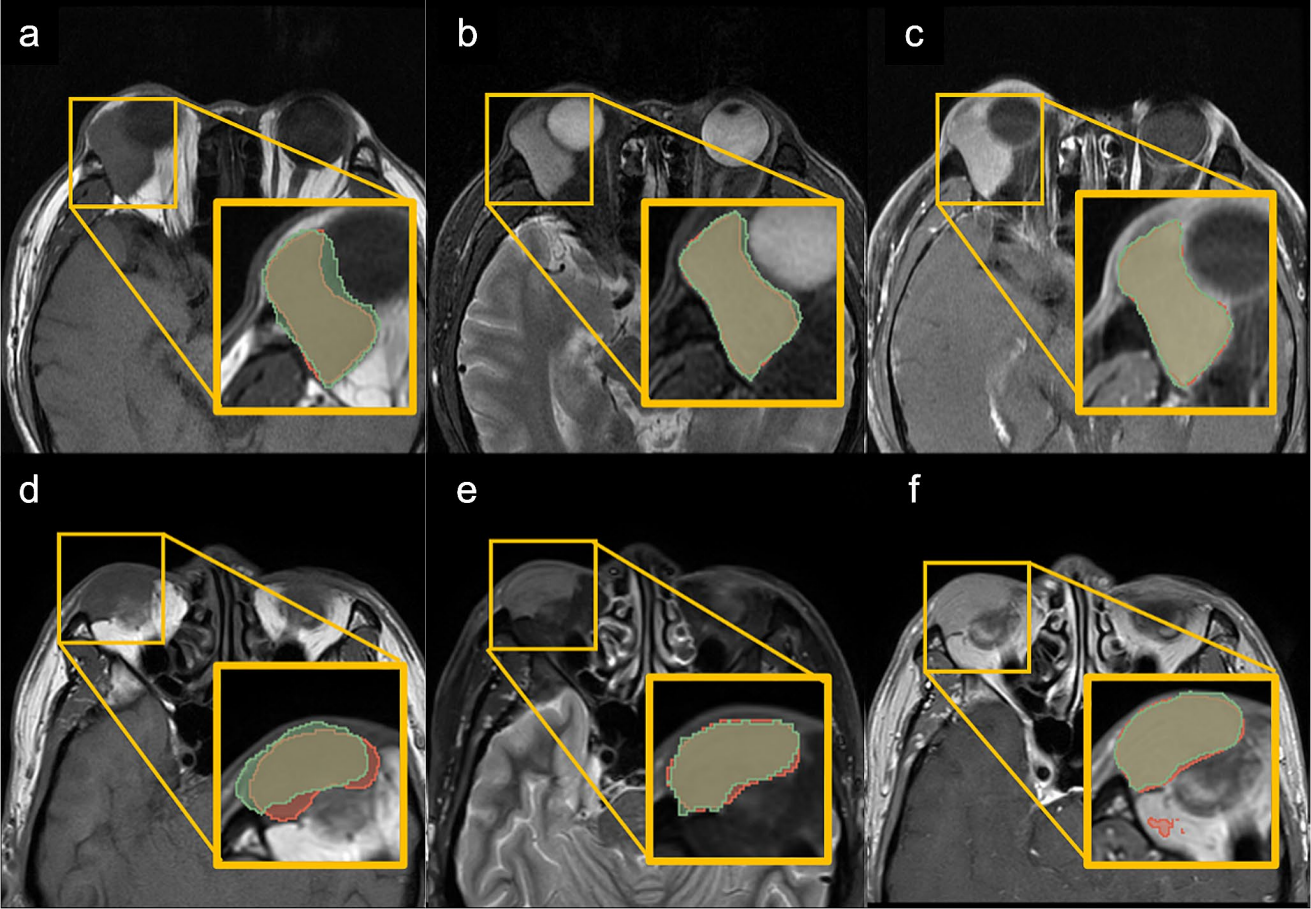
Tumor segmentation performance of OAL with multi-sequence MRI images in Model 1. In the labeled images, the green area represents the manual segmentation, and the red area represents the predicted tumor by nnU-net. **a-c** A 75-year-old male with OAL of the right lacrimal gland. The DSCs of T1 (**a**), T2_FS (**b**), and T1c_FS (**c**) are 0.89, 0.90, and 0.95, respectively. **d-f** A 46-year-old male was diagnosed with OAL of the right superior outer orbit. The DSCs of T1 (**d**), T2_FS (**e**), and T1c_nFS (**f**) are 0.78, 0.84, and 0.89, respectively

In the evaluation of the test set in Model 1 (Table 3), nnU-net demonstrated the highest median DSC of 0.84 (0.70, 0.90) for T1c_FS, slightly outperforming T2_FS with 0.82 (0.75, 0.87). The DSC for T1 was 0.80 (0.75, 0.87), essentially equivalent to T1c_nFS with 0.80 (0.77, 0.85). The nnU-net showed the lowest DSC of 0.79 (0.75, 0.89) for T2_nFS (Fig. 3a). For PPV, the nnU-net achieved better results for T1 and T1c_FS, with values of 92.8% (88.4%, 95.0%) and 92.1% (86.9%, 94.8%), respectively. T2_nFS and T1c_nFS yielded similar PPVs of 90.2%, which were superior to the PPV for T2_FS with 86.1% (74.7%, 93.7%) (Fig. 3b). The nnU-net exhibited the highest sensitivity of 82.3% (67.3%, 87.9%) for T2_nFS, surpassing the sensitivity of T2_FS at 81.2% (73.2%, 87.9%). These values exceeded the sensitivities of 78.7% (66.0%, 86.1%), 76.3% (64.8%, 82.4%), and 72.0% (66.0%, 85.0%) for T1c_FS, T1, and T1c_nFS, respectively (Figs. 3c and 4).

**Table 3:** The segmentation performance of nnu-net in the test set on multi-sequence MRI images

	Model 1			Model 2		
	DSC	PPV (%)	Sensitivity (%)	DSC	PPV (%)	Sensitivity (%)
T1	0.80 (0.75, 0.87)	92.8 (88.4, 95.0)	76.3 (64.8, 82.4)	0.79 (0.74, 0.87)	92.2 (89.8, 95.1)	72.7 (63.0, 81.8)
T2_FS	0.82 (0.75, 0.87)	86.1 (74.7, 93.7)	81.2 (73.2, 87.9)	0.80 (0.59, 0.86)	84.5 (76.1, 93.2)	77.6 (61.4, 83.8)
T2_nFS^&^	0.79 (0.75, 0.89)	90.2 (68.5, 96.0)	82.3 (67.3, 87.9)	0.76 (0.76, 0.90)	91.8 (69.5, 97.4)	84.3 (62.7, 87.8)
T1c_FS	0.84 (0.70, 0.90)	92.1 (86.9, 94.8)	78.7 (66.0, 86.1)	0 (0, 0)	0 (0, 0)	0 (0, 0)
T1c_nFS	0.80 (0.77, 0.85)	90.2 (85.3, 94.4)	72.0 (66.0, 85.0)	0.59 (0.10, 0.85)	91.2 (20.1, 95.5)	51.4 (6.1, 80.2)

DSC: Dice similarity coefficient; PPV: positive prediction value

Data are indicated as medians (interquartile ranges)

Data^&^ are presented as medians (ranges) due to the inclusion of three cases in T2_nFS

### Performance of Model 1 for volumetric measurement

The Bland-Altman plot of nnU-net demonstrated excellent performance, with a mean difference of 1.07 cm^3^ between the predicted tumor volume and the ground truth value (Table 4; Fig. 3d). For smaller lesions with a volume less than 10 cm^3^, the mean difference was 0.27 cm^3^ between nnU-net prediction and the ground truth (Fig. 3d). The CCC of volume between prediction and manual segmentation was 0.90 for the entire test set (Table 4, Supplementary Fig. S3).

**Table 4:** The concordance correlation coefficients (CCCs) and differences in tumor volume between nnu-net predictions and manual segmentation in model 1 and model 2

	Model 1		Model 2	
	Volumetric differences (cm^3^)	CCCs	Volumetric differences (cm^3^)	CCCs
Test set	1.07	0.90	2.99	0.69
Each MRI sequence				
T1	1.45	0.87	2.25	0.85
T2_FS	0.22	0.96	1.24	0.93
T2_nFS	0.03	0.87	0.03	0.85
T1c_FS	0.99	0.86	6.03	0.25
T1c_nFS	2.37	0.87	4.89	0.44

For each sequence, nnU-net achieved a minor difference of 0.03 cm^3^ and 0.22 cm^3^ between the predicted volume and the manual annotation for T2_nFS and T2_FS, respectively, while the maximal difference was 2.37 cm^3^ for T1c_nFS. The variation between nnU-net predicted volumes and manual outlines in T1c_FS and T1 was 0.99 cm^3^ and 1.45 cm^3^, respectively (Table 4, Supplementary Fig. S4). Among all sequences, T2_FS exhibited the highest CCC of volume between prediction and manual delineation, with a value of 0.96 (Fig. 3f). The CCCs on T1, T2_nFS, T1c_FS, and T1c_nFS images were nearly the same, with values of 0.87, 0.87, 0.86, and 0.87, respectively (Table 4, Supplementary Fig. S3).

### Performance of Model 2 for automated segmentation

**Fig. 5:**
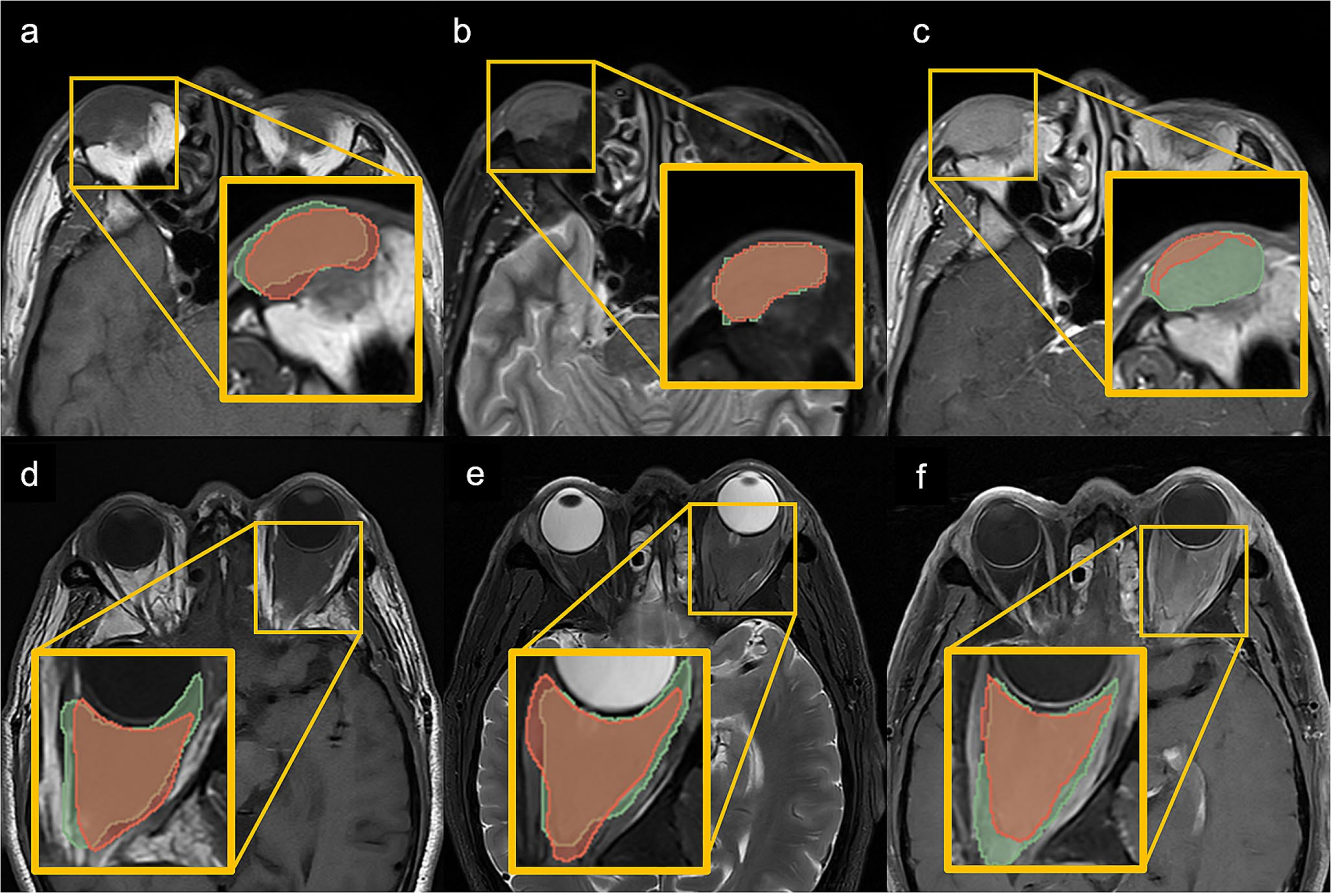
Tumor segmentation performance of OAL with multi-sequence MRI images in Model 2. In the labeled images, the green area represents the manual segmentation, and the red area represents the predicted tumor by nnU-net. **a-c** A 54-year-old female with OAL of the right lacrimal gland. The DSCs of T1 (**a**), T2_FS (**b**), and T1c_nFS (**c**) are 0.77, 0.88, and 0.13, respectively. **d-f** A 70-year-old male was diagnosed with OAL of left space within the muscle cone. The DSCs of T1 (**d**), T2_FS (**e**), and T1c_FS (**f**) are 0.77, 0.79, and 0.77, respectively

We conducted further evaluation of the segmentation performance in Model 2, which contained only non-contrast enhanced T1 and T2 images for training. As shown in Table 3, the DSCs were 0.80 (0.59, 0.86), 0.79 (0.74, 0.87), and 0.76 (0.76, 0.90) for T2_FS, T1 and T2_nFS, respectively (Fig. 3a). However, there were 58% (19/33) cases of T1c that could not be automatically segmented in the test set. Furthermore, nnU-net failed to detect 21% (3/14) cases of T1c_nFS from Center 2, as well as 84% (16/19) cases of T1c_FS (3 cases from Center 2, 4 cases from Center 3, and 9 cases from Center 4). Nonetheless, nnU-net exhibited nearly moderate segmentation performance for T1c_nFS in the remaining 14 cases, achieving a DSC of 0.59 (0.10, 0.85), despite these T1c images not being seen in the training dataset (Table 3; Fig. 3a). The PPV and sensitivity were 92.2% (89.8%, 95.1%) and 72.7% (63.0%, 81.8%), 84.5% (76.1%, 93.2%) and 77.6% (61.4%, 83.8%), 91.8% (69.5%, 97.4%) and 84.3% (62.7%, 87.8%) for T1, T2_FS, and T2_nFS, respectively (Table 3; Fig. 3b-c). For T1c_nFS, the PPV and sensitivity were 91.2% (20.1%, 95.5%) and 51.4% (6.1%, 80.2%), respectively (Table 3; Figs. 3b-c and 5).

### Performance of Model 2 for volumetric measurement

The Bland-Altman plot demonstrated that nnU-net had a mean difference of tumor volume with 2.99 cm^3^ between the prediction and the ground truth in the test set (Table 4; Fig. 3e). The CCC of volume between prediction and ground truth was 0.69 for the entire test set (Table 4, Supplementary Fig. S3).

For each sequence, nnU-net achieved a subtle difference of 0.03 cm^3^ and 1.24 cm^3^ on T2_nFS and T2_FS images, respectively (Table 4, Supplementary Fig. S5). The variation in tumor volume between nnU-net prediction and the ground truth was 2.25 cm^3^, 6.03 cm^3^, and 4.89 cm^3^ for T1, T1c_FS, and T1c_nFS images, respectively (Table 4, Supplementary Fig. S5). The tumor volume predicted by nnU-net moderately matched that outlined by radiologists on T2_FS images, with a CCC of 0.93 (Table 4; Fig. 3f). The CCCs were lower on T1, T2_nFS, T1c_FS and T1c_nFS images with values of 0.85, 0.85, 0.25 and 0.44, respectively. (Table 4, Supplementary Fig. S3).

### Analysis of outliers

Upon analyzing the outliers, we found that nnU-net in Model 1 produced a false-positive region in a small tumor (≤ 10 cm³) on the T2_FS sequence (Fig. S6). For Model 2, nnU-net’s predictions for OAL regions were smaller than the ground truth in two T1c_FS cases (Fig. S7) and larger in the same cases’ T1c_nFS and T2_FS (Fig. S8) images. Notably, the outlier in Model 1 and the overestimated regions in Model 2 were from the same patient, whose lesion was located in the left eyelid. The other two outliers in Model 2 are due to nnU-net predicting false negative regions on T1_FS images. This suggests that in Model 2, nnU-net may be more prone to false negatives on T1_FS images.

## Discussion

In this multi-center study, we have introduced a self-configuring framework based on deep-learning, nnU-net, and have utilized two training models to assess its potential for automatic segmentation and volumetric measurement in OAL. We have further confirmed its generalizability and reproducibility on an external test set with heterogeneous MRI data. The main findings are: (1) Model 1 outperformed Model 2 in segmentation performance across all MRI sequences; (2) nnU-net achieved exceptional performance and reliability in delineating OAL on T2_FS images for both Model 1 and Model 2, with a DSC ranging from 0.80 to 0.82. Additionally, minor discrepancies of 0.22–1.24 cm^3^ in tumor volume between nnU-net predictions and the ground truth were observed on T2_FS, showing acceptable consistency (CCC: 0.93–0.96). These results indicate that nnU-net displays strong adaptability and versatility in delineating and measuring the volume of OAL on multi-sequence MRI datasets, regardless of the imaging equipment and scanning parameters.

Interestingly, despite the absence of T1c_nFS in the training set of Model 1, nnU-net demonstrated great segmentation performance on the test set, achieving a DSC of 0.80 and PPV of 90.2%. Similarly, Model 2’s training set lacked T1c images, yet nnU-net successfully identified the boundaries of OAL in 79% (11/14) of T1c_nFS cases and even in as few as 16% (3/19) of T1c_FS images in the heterogeneous MRI dataset. These findings suggest nnU-net’s ability to identify the boundary of OAL lesions in previously unseen images, further supporting its intrinsic representation learning capability of deep learning. The incorporation of contrast-enhanced images in the training dataset significantly enhanced the scores of DSC for T1c images, with DSCs increasing from 0.59 to 0.80 for T1c_nFS. However, fewer alterations were noted for T1 and T2 images, indicating that a more extensive distribution of the dataset results in improved segmentation performance.

In Model 1, nnU-net yielded the highest DSC with 0.84 for T1c_FS, yet the volumetric difference (0.99 cm^3^vs. 0.22 cm^3^) and CCC (0.86 vs. 0.96) were inferior to T2_FS. Nonetheless, the DSC of T2_FS resembled that of T1c_FS (0.82 vs. 0.84). Although the difference in volume between the nnU-net prediction and manual segmentation on T2_nFS was negligible, at 0.03 cm^3^ for both models. However, this could be influenced by bias originating from the small number of cases (*n* = 3). These findings indicate that T2_FS could be a more appropriate imaging modality for the follow-up of OAL patients, offering meaningful and time-saving benefits for evaluating radiotherapy effectiveness and assisting in optimal treatment selection.

With the advancement of deep learning in the field of radiology, several studies have concentrated on using these methods to select MRI radiomics features and then employing them, such as the conventional neural network, to distinguish between OAL and idiopathic orbital inflammation [26]. Nevertheless, there is limited research exploring the potential of deep learning models for automated segmentation in cases of OAL. The nnU-net’s potential performance for segmenting tumor lesions has gained attention in neuroblastoma [27], lung cancer [28], glioma [29], and meningioma [30]. As far as we know, this marks the initial implementation of nnU-net for automatically segmenting OAL lesions using a multi-sequence MR dataset from various institutions.

Radiotherapy stands as one of the primary treatment approaches for OAL patients due to its widely reported effectiveness [7, 31]. However, many OAL patients typically show residual tumors after radiation therapy [32], which increases the risk of recurrence and necessitates follow-up orbital MRI examinations. The ability to automatically segment OAL lesions on MRI images can significantly aid in monitoring these patients during follow-up. In this present study, we introduced a deep learning-based self-configuring nnU-net network for automatic segmentation of OAL lesions and volume measurement using multi-sequence MRI images. The findings indicated a minimal difference between nnU-net predictions and manual delineations on T2_FS images, implying that nnU-net could serve as an efficient tool for evaluating radiotherapy outcomes in OAL.

In our study, we manually delineated the tumor volumes on each MRI sequence to capture the unique contrast and detail each sequence provides. This approach allowed us to determine that the nnU-net performs best on the T2_FS images, with the smallest difference between the predicted tumor volumes and the manual delineations. While we agree that ultimately there is only one tumor volume, our methodology enabled us to understand how well the algorithm adapts to different MRI contrasts and details. Image registration based on the three MRI sequences and then performing a single delineation is indeed a proposed approach. Currently, our approach of delineating in the original space of each MRI sequence has the advantage of avoiding potential errors introduced by registration. We further evaluated the consistency of the tumor volumes across the three MRI sequences, and our results showed high consistency, which supports the validity of our approach.

The current investigation has several limitations that need to be acknowledged. Firstly, we focused on evaluating the automated segmentation capability of nnU-net specifically for OAL, which resulted in the inclusion of only patients diagnosed with OAL. As a result, there is a shortfall in the discussion concerning the differentiation of OAL from other orbital tumors. We are trying to construct an MRI database of orbital tumors in our country (NCT06336499), and future research will expand upon this foundation by analyzing a larger, more diverse dataset encompassing various types of orbital tumors. Secondly, our assessment of the segmentation performance and volume measurement was restricted to a single deep learning model, nnU-net, thereby overlooking the opportunity to utilize and compare alternative models. In future work, we plan to conduct a broader comparative study encompassing multiple methods to provide a more comprehensive analysis for aiding in clinical method selection. Thirdly, the findings of this study are still in the preliminary stage due to the diversity of MRI data considered. To further validate the feasibility of nnU-net in OAL segmentation, it is essential to carry out studies with larger sample sizes and incorporate more diverse MRI data types, such as diffusion-weighted imaging (DWI), apparent diffusion coefficient (ADC) maps, and dynamic contrast-enhanced MRI (DCE-MRI). Finally, due to the relatively small sample size of the external test set, we cannot definitively determine common causes for the outliers in each model. This indicates the need to increase the sample size in future research for a more thorough investigation and summary. Nevertheless, we remain hopeful that the analysis of typical cases identified among outliers will offer valuable insights for future research or for anyone attempting to replicate the proposed method.

## Conclusions

In summary, nnU-net offered excellent performance and robustness in the automated segmentation and volumetric evaluation of routine MRI images for OAL patients, particularly on T2_FS images. This suggests its promising potential for assessing therapeutic efficacy and streamlining the follow-up workflow. Besides, nnU-net demonstrates the ability to predict the boundaries of OAL tumors on T1c images trained by unseen contrast-enhanced MRI images, which reduces the requirement for manual delineation and minimizes the duration of model training.

## Electronic supplementary material

Below is the link to the electronic supplementary material.

Supplementary Material 1

## Data Availability

The data collected for this research is accessible upon reasonable request to the corresponding author.

## Code Availability

The original code of nnU-net is available in the GitHub repository (https://github.com/mic-dkfz/nnunet).

## References

[1] Kirkegaard MK (2022) Ocular adnexal lymphoma: subtype-specific clinical and genetic features. Acta Ophthalmol 100 Suppl 270:3–37 10.1111/aos.15248 36196757

[2] Yen MT, Bilyk JR, Wladis EJ, Bradley EA, Mawn LA (2018) Treatments for ocular adnexal lymphoma: a report by the American Academy of Ophthalmology. Ophthalmology 125:127–136 28712656 10.1016/j.ophtha.2017.05.037

[3] Loya A, Ramachandran V, Ayaz T, Weng CY (2021) Second primary malignancies after ocular adnexal lymphoma diagnosis. BMC Ophthalmol 21:162 33827494 10.1186/s12886-021-01921-7 PMC8028208

[4] Darwich R, Ghazawi FM, Rahme E, Alghazawi N, Zubarev A, Moreau L, Sasseville D, Burnier MN Jr., Litvinov IV (2020) Epidemiology of ophthalmic lymphoma in Canada during 1992–2010. Br J Ophthalmol 104:1176–1180 31722877 10.1136/bjophthalmol-2019-314653

[5] Olsen TG, Heegaard S (2019) Orbital lymphoma. Surv Ophthalmol 64:45–66 30144455 10.1016/j.survophthal.2018.08.002

[6] Holm F, Mikkelsen LH, Kamper P, Rasmussen PK, Larsen TS, Sjö LD, Heegaard S (2021) Ocular adnexal lymphoma in Denmark: a nationwide study of 387 cases from 1980 to 2017. Br J Ophthalmol 105:914–920 32732342 10.1136/bjophthalmol-2019-315637

[7] Rehn S, Elsayad K, Oertel M, Baehr A, Eter N, Haverkamp U, Lenz G, Eich HT (2020) Radiotherapy Dose and volume de-escalation in Ocular Adnexal Lymphoma. Anticancer Res 40:4041–4046 32620650 10.21873/anticanres.14400

[8] Yang X, Wang R, Yuan X, Yao S, Wang C, Cheng J (2022) Ultra-low-dose radiotherapy in the treatment of ocular adnexal lymphoma: a prospective study. Radiat Oncol 17:208 36539787 10.1186/s13014-022-02180-6 PMC9764465

[9] Pereira-Da Silva MV, Di Nicola ML, Altomare F, Xu W, Tsang R, Laperriere N, Krema H (2023) Radiation therapy for primary orbital and ocular adnexal lymphoma. Clin Transl Radiat Oncol 38:15–20 36353653 10.1016/j.ctro.2022.10.001 PMC9637715

[10] Unkelbach J, Bortfeld T, Cardenas CE, Gregoire V, Hager W, Heijmen B, Jeraj R, Korreman SS, Ludwig R, Pouymayou B, Shusharina N, Söderberg J, Toma-Dasu I, Troost EGC, Vasquez Osorio E (2020) The role of computational methods for automating and improving clinical target volume definition. Radiother Oncol 153:15–25 33039428 10.1016/j.radonc.2020.10.002

[11] Almeida G, Tavares J (2020) Deep learning in Radiation Oncology Treatment planning for prostate Cancer: a systematic review. J Med Syst 44:179 32862251 10.1007/s10916-020-01641-3

[12] Otazo R, Lambin P, Pignol JP, Ladd ME, Schlemmer HP, Baumann M, Hricak H (2021) MRI-guided Radiation Therapy: an emerging paradigm in adaptive Radiation Oncology. Radiology 298:248–260 33350894 10.1148/radiol.2020202747 PMC7924409

[13] Moore-Palhares D, Ho L, Lu L, Chugh B, Vesprini D, Karam I, Soliman H, Symons S, Leung E, Loblaw A, Myrehaug S, Stanisz G, Sahgal A, Czarnota GJ (2023) Clinical implementation of magnetic resonance imaging simulation for radiation oncology planning: 5 year experience. Radiat Oncol 18:27 36750891 10.1186/s13014-023-02209-4 PMC9903411

[14] Lecler A, Duron L, Charlson E, Kolseth C, Kossler AL, Wintermark M, Moulin K, Rutt B (2022) Comparison between 7 Tesla and 3 Tesla MRI for characterizing orbital lesions. Diagn Interv Imaging 103:433–439 35410799 10.1016/j.diii.2022.03.007 PMC12498293

[15] Fei Y, Zhan B, Hong M, Wu X, Zhou J, Wang Y (2021) Deep learning-based multi-modal computing with feature disentanglement for MRI image synthesis. Med Phys 48:3778–3789 33959965 10.1002/mp.14929

[16] Xiang Y, Zeng C, Liu B, Tan W, Wu J, Hu X, Han Y, Luo Q, Gong J, Liu J, Li Y (2022) Deep learning-enabled identification of autoimmune encephalitis on 3D Multi-sequence MRI. J Magn Reson Imaging 55:1082–1092 34478565 10.1002/jmri.27909

[17] Xia Y, Ravikumar N, Lassila T, Frangi AF (2023) Virtual high-resolution MR Angiography from non-angiographic multi-contrast MRIs: synthetic vascular model populations for in-silico trials. Med Image Anal 87:102814 37196537 10.1016/j.media.2023.102814

[18] Isensee F, Jaeger PF, Kohl SAA, Petersen J, Maier-Hein KH (2021) nnU-Net: a self-configuring method for deep learning-based biomedical image segmentation. Nat Methods 18:203–211 33288961 10.1038/s41592-020-01008-z

[19] Gibson E, Li W, Sudre C, Fidon L, Shakir DI, Wang G, Eaton-Rosen Z, Gray R, Doel T, Hu Y, Whyntie T, Nachev P, Modat M, Barratt DC, Ourselin S, Cardoso MJ, Vercauteren T (2018) NiftyNet: a deep-learning platform for medical imaging. Comput Methods Programs Biomed 158:113–122 29544777 10.1016/j.cmpb.2018.01.025 PMC5869052

[20] Doss DJ, Johnson GW, Narasimhan S, Shless JS, Jiang JW, González HFJ, Paulo DL, Lucas A, Davis KA, Chang C, Morgan VL, Constantinidis C, Dawant BM, Englot DJ (2023) Deep learning segmentation of the Nucleus Basalis of meynert on 3T MRI. AJNR Am J Neuroradiol 44:1020–1025 37562826 10.3174/ajnr.A7950 PMC10494939

[21] Cuocolo R, Comelli A, Stefano A, Benfante V, Dahiya N, Stanzione A, Castaldo A, De Lucia DR, Yezzi A, Imbriaco M (2021) Deep learning whole-gland and zonal prostate segmentation on a public MRI dataset. J Magn Reson Imaging 54:452–459 33634932 10.1002/jmri.27585

[22] Sengupta PP, Shrestha S, Berthon B, Messas E, Donal E, Tison GH, Min JK, D’Hooge J, Voigt JU, Dudley J, Verjans JW, Shameer K, Johnson K, Lovstakken L, Tabassian M, Piccirilli M, Pernot M, Yanamala N, Duchateau N, Kagiyama N, Bernard O, Slomka P, Deo R, Arnaout R (2020) Proposed requirements for Cardiovascular Imaging-Related machine learning evaluation (PRIME): a checklist: reviewed by the American College of Cardiology Healthcare Innovation Council. JACC Cardiovasc Imaging 13:2017–2035 32912474 10.1016/j.jcmg.2020.07.015 PMC7953597

[23] Zhou W, Yang Y, Yu C, Liu J, Duan X, Weng Z, Chen D, Liang Q, Fang Q, Zhou J, Ju H, Luo Z, Guo W, Ma X, Xie X, Wang R, Zhou L (2021) Ensembled deep learning model outperforms human experts in diagnosing biliary atresia from sonographic gallbladder images. Nat Commun 12:1259 33627641 10.1038/s41467-021-21466-z PMC7904842

[24] McBride G (2005) A proposal for strength-of-agreement criteria for Lin’s concordance correlation coefficient. NIWA Client Report: HAM2005-062 45:307–310

[25] Bischoff LM, Peeters JM, Weinhold L, Krausewitz P, Ellinger J, Katemann C, Isaak A, Weber OM, Kuetting D, Attenberger U, Pieper CC, Sprinkart AM, Luetkens JA (2023) Deep Learning Super-resolution Reconstruction for fast and motion-robust T2-weighted prostate MRI. Radiology 308:e230427 37750774 10.1148/radiol.230427

[26] Xie X, Yang L, Zhao F, Wang D, Zhang H, He X, Cao X, Yi H, He X, Hou Y (2022) A deep learning model combining multimodal radiomics, clinical and imaging features for differentiating ocular adnexal lymphoma from idiopathic orbital inflammation. Eur Radiol 32:6922–6932 35674824 10.1007/s00330-022-08857-6

[27] Veiga-Canuto D, Cerdà-Alberich L, Jiménez-Pastor A, Carot Sierra JM, Gomis-Maya A, Sangüesa-Nebot C, Fernández-Patón M, Martínez de Las Heras B, Taschner-Mandl S, Düster V, Pötschger U, Simon T, Neri E, Alberich-Bayarri Á, Cañete A, Hero B, Ladenstein R, Martí-Bonmatí L (2023) Independent validation of a Deep Learning Nnu-Net Tool for Neuroblastoma Detection and Segmentation in MR images. Cancers (Basel) 15 10.3390/cancers15051622 PMC10000775 36900410

[28] Zhang G, Yang Z, Huo B, Chai S, Jiang S (2021) Automatic segmentation of organs at risk and tumors in CT images of lung cancer from partially labelled datasets with a semi-supervised conditional nnu-net. Comput Methods Programs Biomed 211:106419 34563895 10.1016/j.cmpb.2021.106419

[29] Verdier M, Deverdun J, de Champfleur NM, Duffau H, Lam P, Santos TD, Troalen T, Maréchal B, Huelnhagen T, Bars EL (2023) Evaluation of a nnu-net type automated clinical volumetric tumor segmentation tool for diffuse low-grade glioma follow-up. J Neuroradiol. 10.1016/j.neurad.2023.05.008 37308338 10.1016/j.neurad.2023.05.008

[30] Kang H, Witanto JN, Pratama K, Lee D, Choi KS, Choi SH, Kim KM, Kim MS, Kim JW, Kim YH, Park SJ, Park CK (2023) Fully automated MRI segmentation and volumetric measurement of Intracranial Meningioma using deep learning. J Magn Reson Imaging 57:871–881 35775971 10.1002/jmri.28332

[31] Zhou M, Wang J, Shi J, Zhai G, Zhou X, Ye L, Li L, Hu M, Zhou Y (2024) Prediction model of radiotherapy outcome for ocular adnexal lymphoma using informative features selected by chemometric algorithms. Comput Biol Med 170:108067 38301513 10.1016/j.compbiomed.2024.108067

[32] Hoffmann C, Mohr C, Johansson P, Eckstein A, Huettmann A, von Tresckow J, Göricke S, Deuschl C, Poettgen C, Gauler T, Guberina N, Moliavi S, Bechrakis N, Stuschke M, Guberina M (2023) MRI-based long-term follow-up of indolent orbital lymphomas after curative radiotherapy: imaging remission criteria and volumetric regression kinetics. Sci Rep 13:4792 36959374 10.1038/s41598-023-31941-w PMC10036339

