# Correction to: Fully automated segmentation and volumetric measurement of ocular adnexal lymphoma by deep learning-based self-configuring nnU-net on multi-sequence MRI: a multi-center study

**DOI:** 10.1007/s00234-024-03437-5

**Published:** 2024-08-01

**Authors:** Guorong Wang, Bingbing Yang, Xiaoxia Qu, Jian Guo, Yongheng Luo, Xiaoquan Xu, Feiyun Wu, Xiaoxue Fan, Yang Hou, Song Tian, Sicong Huang, Junfang Xian

**Affiliations:** 1 Department of Radiology, Beijing Tongren Hospital, Capital Medical University, No.1 DongJiaoMinXiang Street, DongCheng District, Beijing, 100730 China; 2 Department of Radiology, The Second Xiangya Hospital, Central South University, Changsha, China; 3 Department of Radiology, The First Affiliated Hospital of Nanjing Medical University, Nanjing, China; 4 Department of Radiology, Shengjing Hospital of China Medical University, Shenyang, China; 5 Philips Healthcare, Beijing, China

**Correction to: Neuroradiology (2024)**

10.1007/s00234-024-03429-5

The original version of this article unfortunately contained a mistake.

Figures 3–5 has been interchanged. To clarify, the correct order should be as follows:

- The current Fig. 4 should be moved to the position of Fig. 3.
- The current Fig. 5 should be moved to the position of Fig. 4.
- The current Fig. 3 should be moved to the position of Fig. 5.

The original article has been corrected.

