# Supplementary material for: Fully automated segmentation and volumetric measurement of ocular adnexal lymphoma by deep learning-based self-configuring nnU-net on multi-sequence MRI: a multi-center study
Source: Neuroradiology. 2024 Jul 17;66(10):1781–91. doi: 10.1007/s00234-024-03429-5 (PMC11424727; doi:10.1007/s00234-024-03429-5)

**Fully automated segmentation and volumetric measurement of ocular adnexal lymphoma by deep learning-based self-configuring nnU-net on multi-sequence MRI: a multi-center study**

**Supplementary materials**

1. **Supplementary tables**

**Table S1.** MRI scan parameters of each center

| Center 1 |  |  |  |
| --- | --- | --- | --- |
| 3T |  |  |  |
| Parameters | T1 | T2 | T1c |
| Acquisition matrix | 320-512×320-512 | 384-512×384-512 | 320-800×384-800 |
| Slice thickness (mm) | 3-3.5 | 3-3.5 | 3-3.5 |
| Repetition time (msec) | 400-758 | 1936.4-3798 | 357-887 |
| Echo time (msec) | 8.5-11 | 84-120.8 | 7.7-12 |
| 1.5T |  |  |  |
| Parameters | T1 | T2 | T1c |
| Acquisition matrix | 384×384 | 384×384 | 384×384 |
| Slice thickness (mm) | 3 | 3 | 3 |
| Repetition time (msec) | 550 | 3000 | 585.6 |
| Echo time (msec) | 15 | 120 | 16 |
| Center 2 |  |  |  |
| 3T |  |  |  |
| Parameters | T1 | T2 | T1c |
| Acquisition matrix | 192-640×192-640 | 320-768×320-768 | 192-640×192-640 |
| Slice thickness (mm) | 2.5-4 | 2.5-3 | 2.5-3 |
| Repetition time (msec) | 446-700 | 2912-4030 | 445-622 |
| Echo time (msec) | 6.4-10 | 79.3-117 | 6.7-9.7 |
| Center 3 |  |  |  |
| 3T |  |  |  |
| Parameters | T1 | T2 | T1c |
| Acquisition matrix | 336-576×336-576 | 240-400×240-400 | 384-576×384-576 |
| Slice thickness (mm) | 3-4 | 3-4 | 3-4 |
| Repetition time (msec) | 485.4-566.3 | 2500-3000 | 450-605 |
| Echo time (msec) | 7-14 | 80-90 | 7.5-7 |
| Center 4 |  |  |  |
| 3T |  |  |  |
| Parameters | T1 | T2 | T1c |
| Acquisition matrix | 320-432×320-432 | 320-432×320-432 | 256-384×256-384 |
| Slice thickness (mm) | 2.5-3.5 | 2.5-3 | 2.5-3.5 |
| Repetition time (msec) | 400-625 | 3134-4800 | 285-561 |
| Echo time (msec) | 9-11 | 75-92 | 10.7-11 |

**Table S2. Segmentation performance of training set in Model 1**

|  | DSC | PPV (%) | Sensitivity (%) | Volumetric  Difference (cm^3^) | CCC |
| --- | --- | --- | --- | --- | --- |
| Fold_0 |  |  |  |  |  |
| T1 | 0.80 (0.65, 0.84) | 81.9 (59.5, 89.7) | 79.0 (67.8, 86.4) | 0.25 | 0.98 |
| T2_FS | 0.87 (0.83, 0.89) | 88.3 (85.4, 90.0) | 87.3 (83.0, 90.6) | 0.20 | 0.99 |
| T2_nFS | 0.78 (0.67, 0.88) | 75.9 (60.6, 92.6) | 81.0 (77.2, 90.9) | 0.15 | 0.86 |
| T1c_FS | 0.86 (0.80, 0.90) | 87.2 (76.7, 91.8) | 87.0 (82.8, 92.1) | 0.18 | 0.98 |
| Fold_1 |  |  |  |  |  |
| T1 | 0.83 (0.72, 0.88) | 84.3 (79.0, 91.2) | 79.6 (70.7, 88.5) | 0.35 | 0.99 |
| T2_FS | 0.83 (0.68, 0.90) | 91.4 (85.6, 92.7) | 82.7 (55.4,88.6) | 1.08 | 0.98 |
| T2_nFS | 0.86 (0.83, 0.88) | 85.5 (82.3, 89.8) | 87.3 (81.5, 89.1) | 0.17 | 0.99 |
| T1c_FS | 0.85 (0.77, 0.90) | 84.2 (71.6, 86.4) | 91.5 (77.4, 94.0) | 0.47 | 0.96 |
| Fold_2 |  |  |  |  |  |
| T1 | 0.81 (0.72, 0.87) | 82.7 (73.3, 87.7) | 83.7 (69.1, 89.8) | 0.02 | 0.96 |
| T2_FS | 0.87 (0.79, 0.91) | 87.6 (82.8, 88.9) | 88.8 (83.8, 91.6) | 0.14 | 0.94 |
| T2_nFS | 0.80 (0.72, 0.88) | 87.5 (77.8, 90.9) | 83.0 (63.4, 88.9) | 0.58 | 0.95 |
| T1c_FS | 0.85 (0.82, 0.91) | 89.1 (83.3, 94.3) | 84.0 (76.0, 91.2) | 1.28 | 0.88 |
| Fold_3 |  |  |  |  |  |
| T1 | 0.80 (0.73, 0.85) | 83.1 (78.9, 91.9) | 80.7 (67.4, 86.8) | 0.28 | 0.97 |
| T2_FS | 0.81 (0.75, 0.87) | 87.4 (73.6, 92.3) | 78.8 (71.2, 87.1) | 0.17 | 0.94 |
| T2_nFS | 0.79 (0.74, 0.84) | 82.5 (72.8, 87.2) | 82.4 (71.2, 88.7) | 0.44 | 0.89 |
| T1c_FS | 0.86 (0.71, 0.89) | 86.0 (80.6, 90.0) | 86.8 (73.7, 92.5) | 0.42 | 0.92 |
| Fold_4 |  |  |  |  |  |
| T1 | 0.84 (0.76, 0.88) | 86.0 (79.8, 88.5) | 83.3 (77.2, 89.0) | 0.73 | 0.90 |
| T2_FS | 0.85 (0.63, 0.92) | 82.3 (70.2, 93.1) | 88.1 (58.2, 91.5) | 0.35 | 0.98 |
| T2_nFS | 0.83 (0.68, 0.87) | 84.0 (76.6, 88.4) | 81.3 (67.1, 85.8) | 0.80 | 0.96 |
| T1c_FS | 0.82 (0.68, 0.86) | 82.0 (74.6, 89.3) | 80.8 (71.0, 90.7) | 0.37 | 0.97 |

**Table S3. Segmentation performance of training set in Model 2**

|  | DSC | PPV (%) | Sensitivity (%) | Volumetric  Difference (cm^3^) | CCC |
| --- | --- | --- | --- | --- | --- |
| Fold_0 |  |  |  |  |  |
| T1 | 0.81 (0.68, 0.87) | 86.2 (76.3, 90.5) | 78.7 (65.1, 86.6) | 1.01 | 0.88 |
| T2_FS | 0.86 (0.75, 0.89) | 91.5 (84.9, 93.0) | 84.2 (78.8, 86.3) | 0.59 | 0.93 |
| T2_nFS | 0.75 (0.66, 0.86) | 82.2 (75.2, 91.8) | 73.3 (58.8, 86.1) | 1.71 | 0.89 |
| Fold_1 |  |  |  |  |  |
| T1 | 0.83 (0.77, 0.87) | 85.0 (73.8, 91.6) | 82.5 (73.6, 88.2) | 0.16 | 0.99 |
| T2_FS | 0.86 (0.73, 0.88) | 90.2 (80.0, 92.0) | 82.1 (62.3, 85.9) | 0.80 | 0.99 |
| T2_nFS | 0.81 (0.69, 0.87) | 88.2 (81.1, 91.2) | 80.0 (58.0, 83.6) | 0.89 | 0.91 |
| Fold_2 |  |  |  |  |  |
| T1 | 0.82 (0.74, 0.86) | 84.1 (77.5, 89.4) | 83.6 (71.4, 87.4) | 0.17 | 0.97 |
| T2_FS | 0.79 (0.48, 0.87) | 88.8 (83.7, 94.8) | 77.1 (45.2, 86.5) | 0.43 | 0.90 |
| T2_nFS | 0.82 (0.77, 0.89) | 88.1 (71.5, 92.3) | 84.5 (73.9, 89.3) | 0.08 | 0.96 |
| Fold_3 |  |  |  |  |  |
| T1 | 0.82 (0.66, 0.89) | 87.3 (73.0, 91.5) | 85.0 (68.2, 89.3) | 0.10 | 0.91 |
| T2_FS | 0.81 (0.67, 0.87) | 77.9 (67.5, 88.6) | 86.1 (72.3, 91.0) | 0.45 | 0.84 |
| T2_nFS | 0.81 (0.72, 0.88) | 81.4 (66.6, 88.8) | 81.9 (71.7, 92.0) | 0.25 | 0.98 |
| Fold_4 |  |  |  |  |  |
| T1 | 0.80 (0.73, 0.84) | 79.7 (73.1, 87.5) | 81.6 (68.5, 86.1) | 0.31 | 0.98 |
| T2_FS | 0.81 (0.70, 0.88) | 83.5 (73.6, 88.9) | 76.4 (62.6, 89.5) | 0.42 | 0.95 |
| T2_nFS | 0.82 (0.79, 0.89) | 87.8 (79.3, 91.1) | 82.6 (78.0, 90.0) | 0.28 | 0.98 |

1. **Supplementary figures**

**Fig. S1** Schematic framework of the 5-fold cross validation process during data training.

**
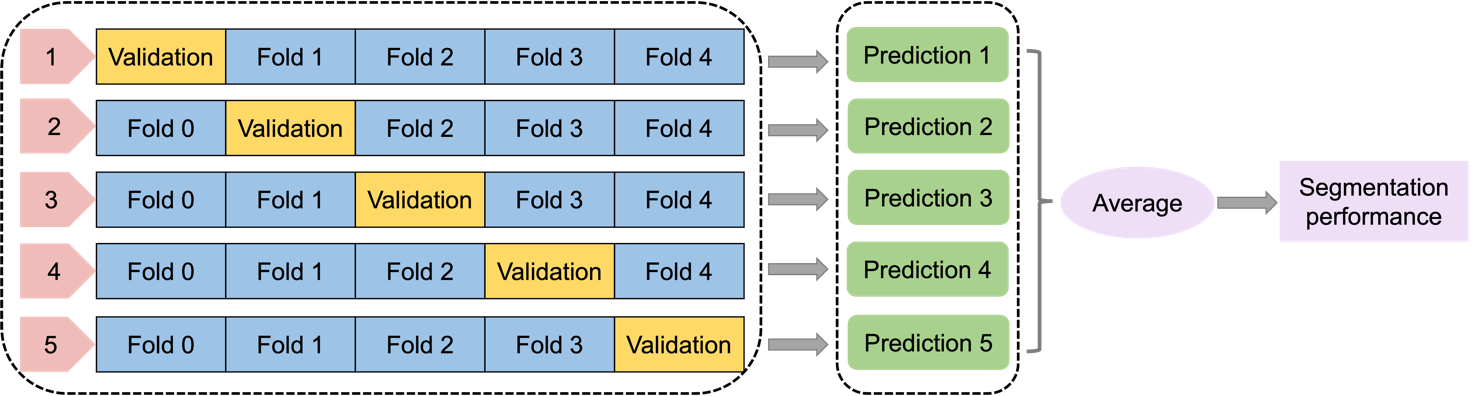
**

**Fig. S2** The DSC **(a)**, PPV**(b)**, sensitivity **(c)**, and CCC **(d)** of training set in Model 1 and 2.


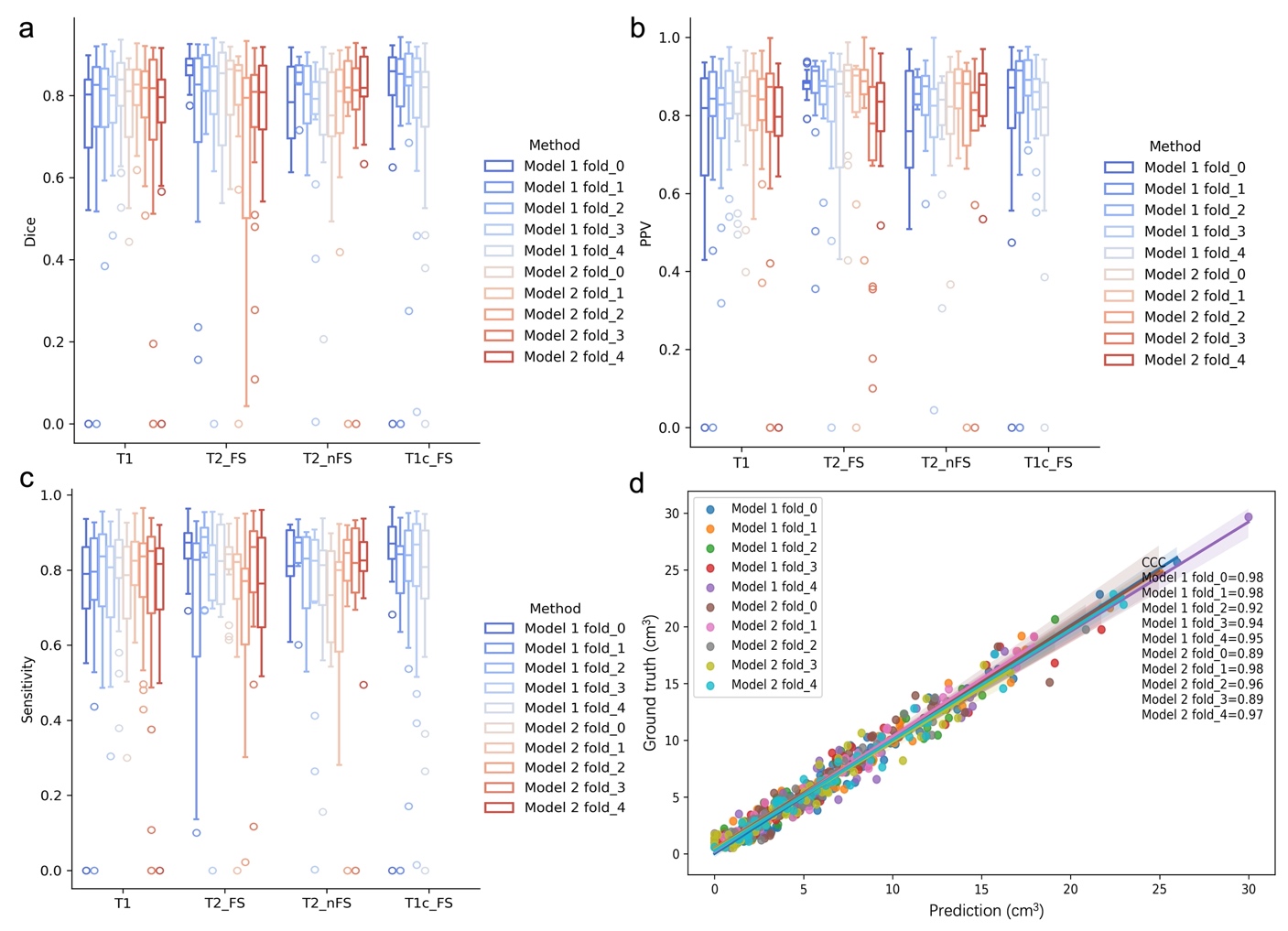


**Fig. S3** The CCCs of the test set **(a)**, T1 **(b)**, T2_nFS **(c)**, T1c_FS **(d)**, and T1c_nFS **(e)** images in Model 1 and Model 2.


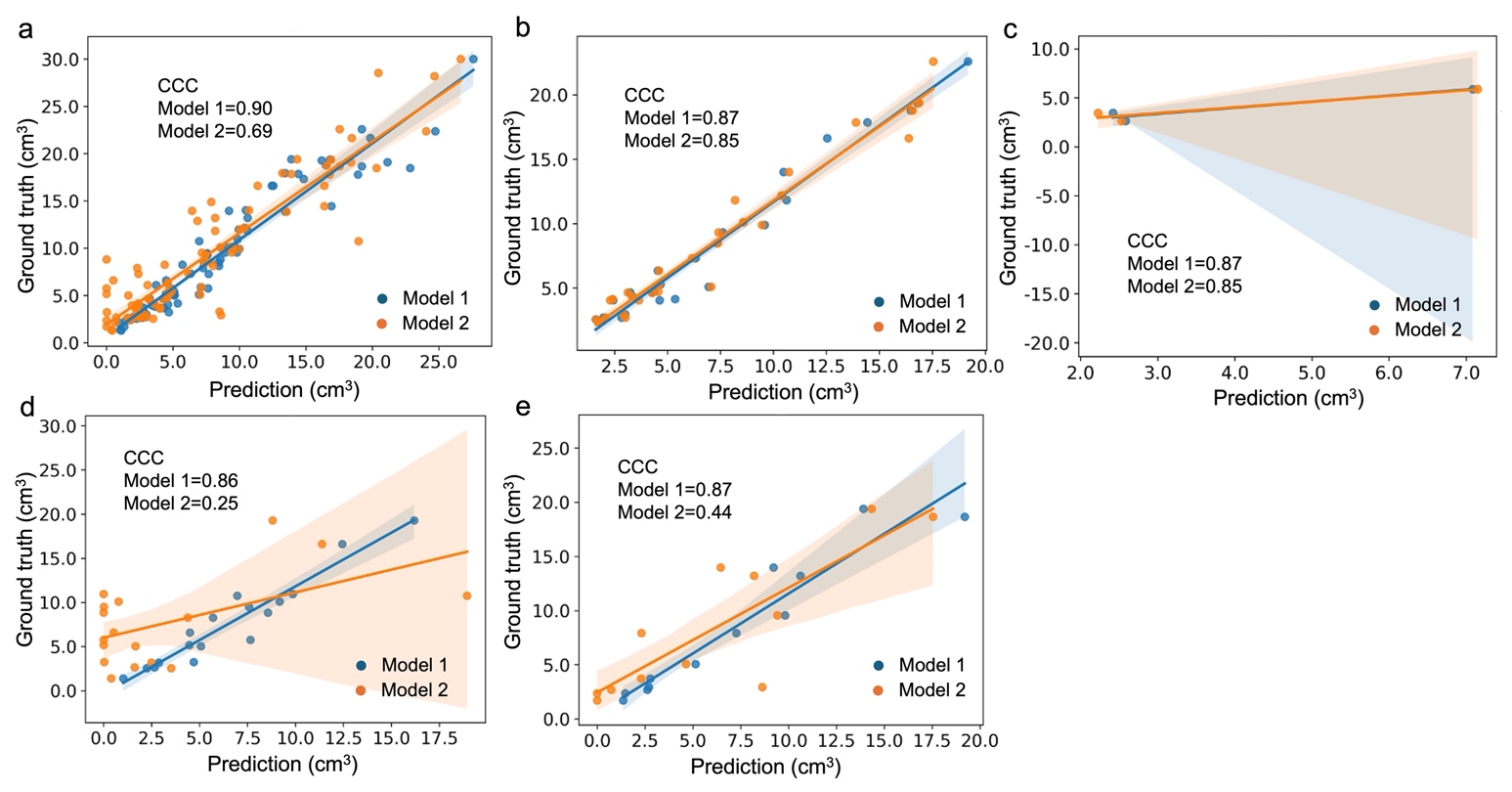


**Fig. S4** The Bland-Altman plots of T1 **(a)**, T2_FS **(b)**, T2_nFS **(c)**, T1c_FS **(d)**, and T1c_nFS **(e)** images in Model 1.

**
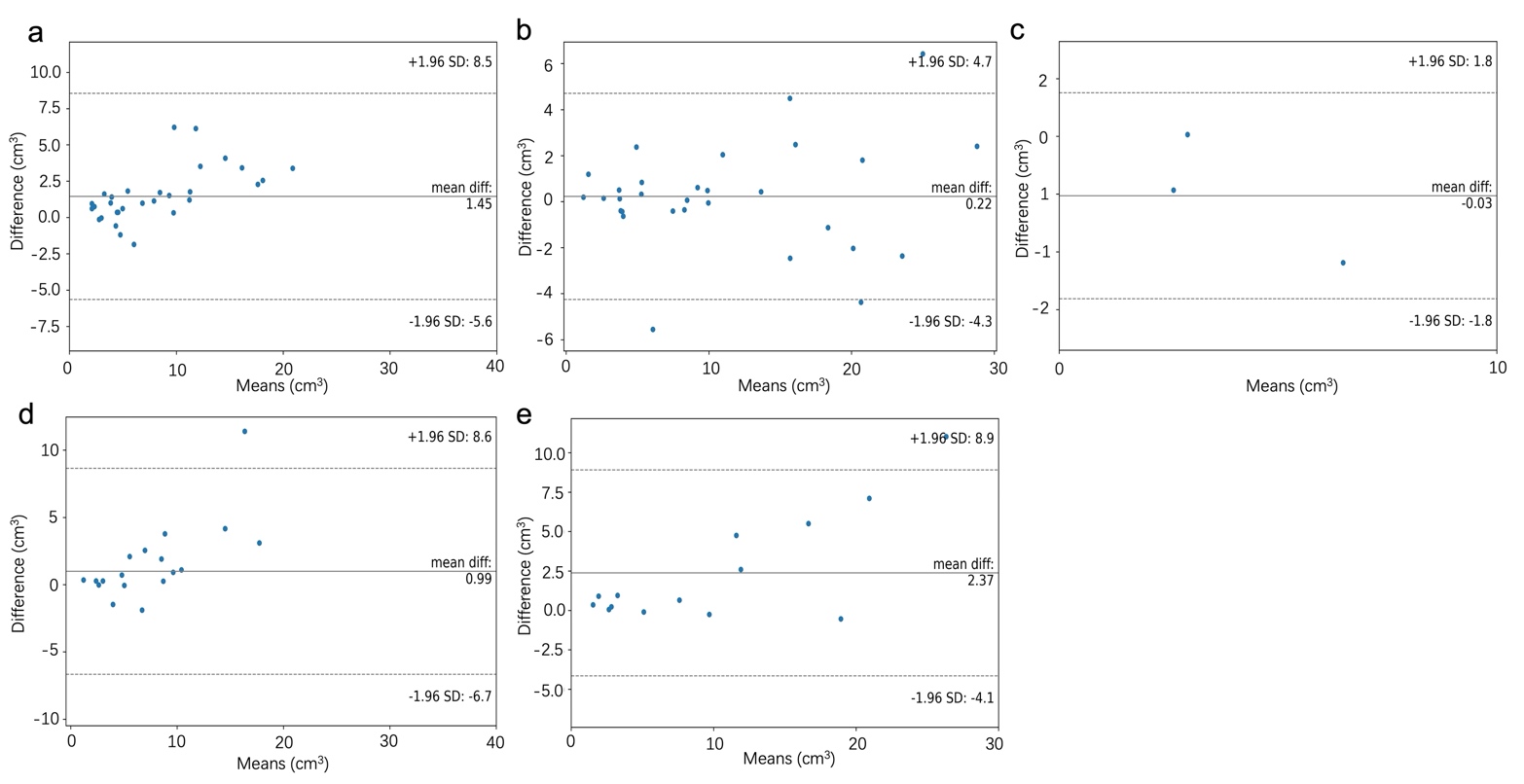
**

**Fig. S5** The Bland-Altman plots of T1 **(a)**, T2_FS **(b)**, T2_nFS **(c)**, T1c_FS **(d)**, and T1c_nFS **(e)** images in Model 2.

**
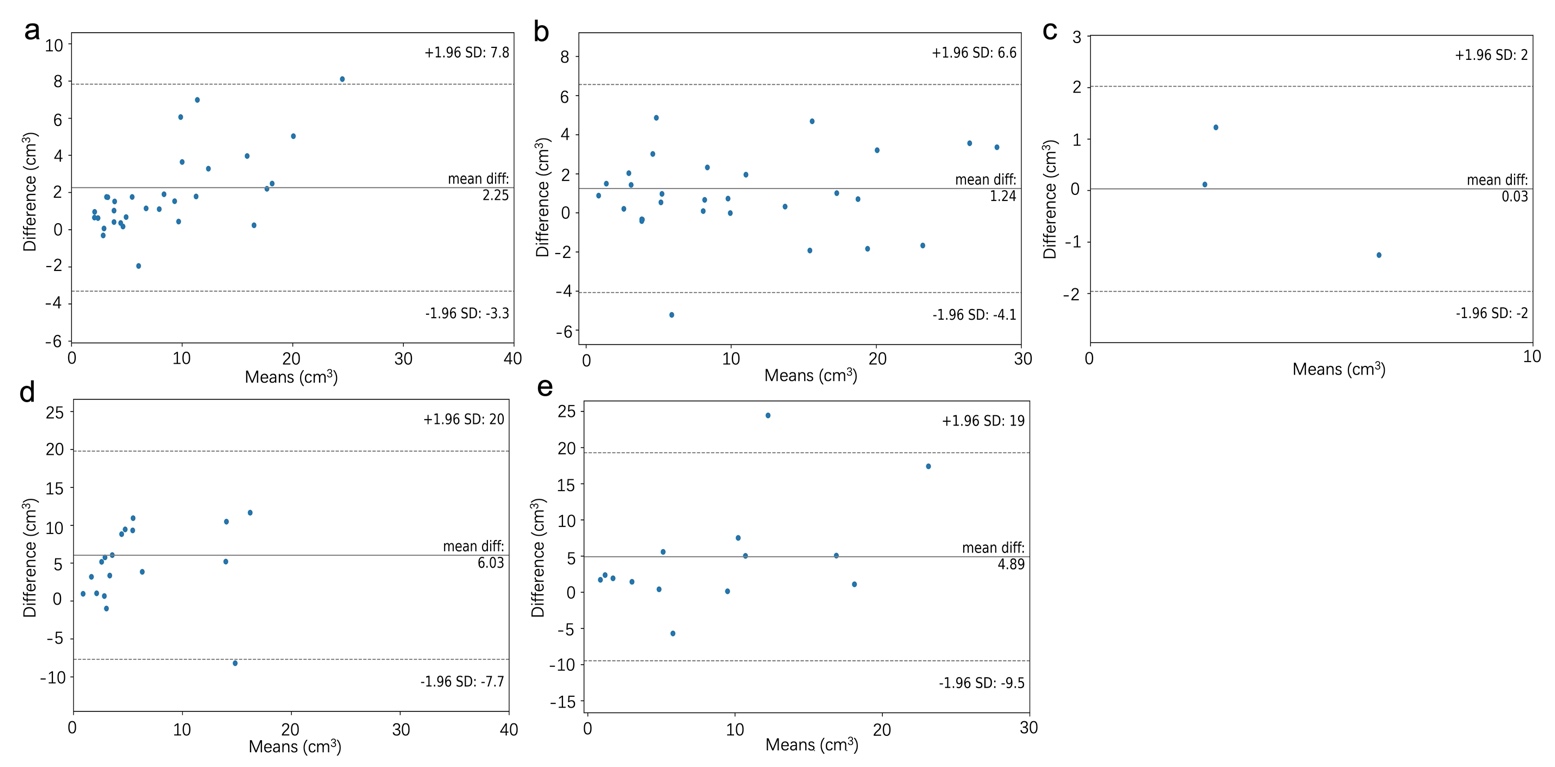
**

**Fig. S6** The nnU-net predicted OAL on T2_FS sequence (a) and its corresponding 3D display image (b) for Model 1. Red: ground truth; Green: nnU-net prediction; Yellow: merged area. The green area displayed separately after merging represents the false-positive region identified by nnU-net.

**
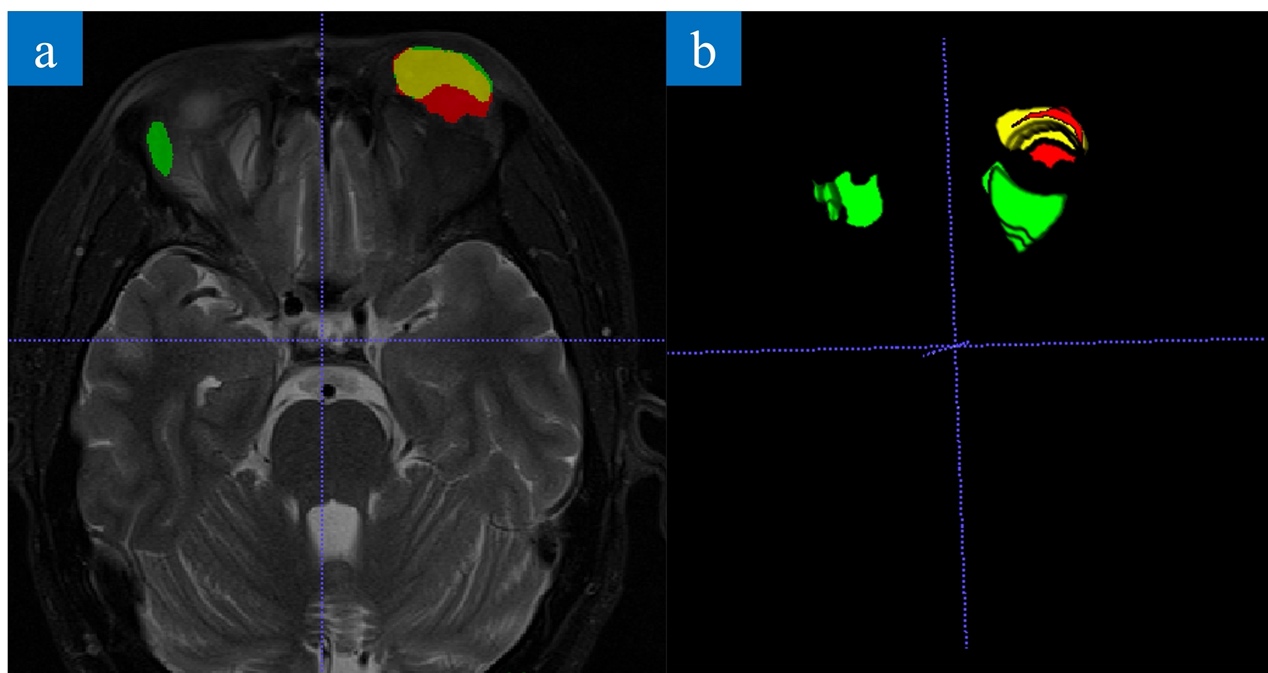
**

**Fig. S7** Two cases of nnU-net predicted OAL on T1c_FS sequence (a, c) and its corresponding 3D display images (b, d) for Model 2. Red: ground truth; Green: nnU-net prediction; Yellow: merged area. The red area displayed separately after merging represents the false-negative region identified by nnU-net.


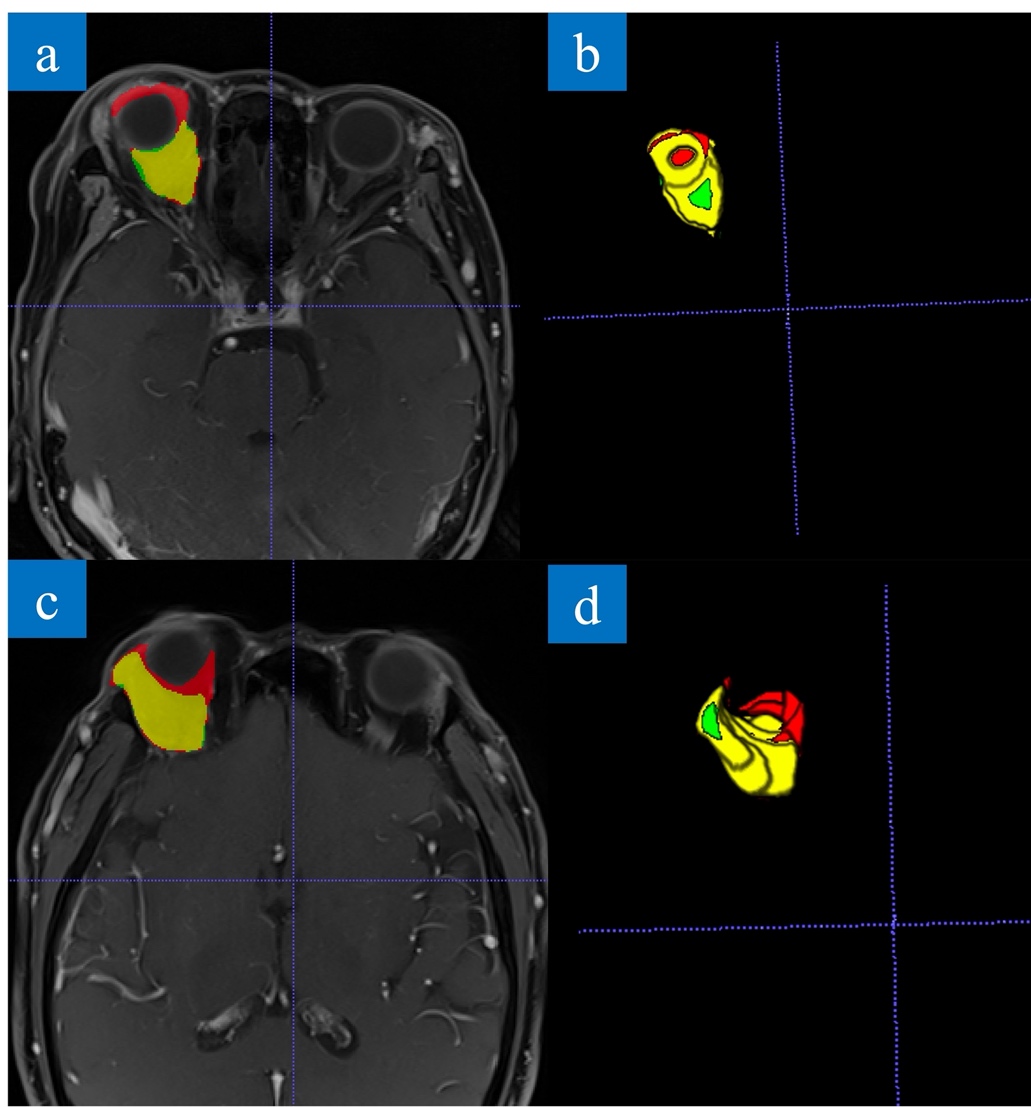


**Fig. S8** The nnU-net predicted OAL on T1c_nFS (a-b) and T2_FS sequences (d-e) for Model 2, with corresponding 3D display images (c, f). Red: ground truth; Green: nnU-net prediction; Yellow: merged area. The green area displayed separately after merging represents the false-positive region identified by nnU-net.


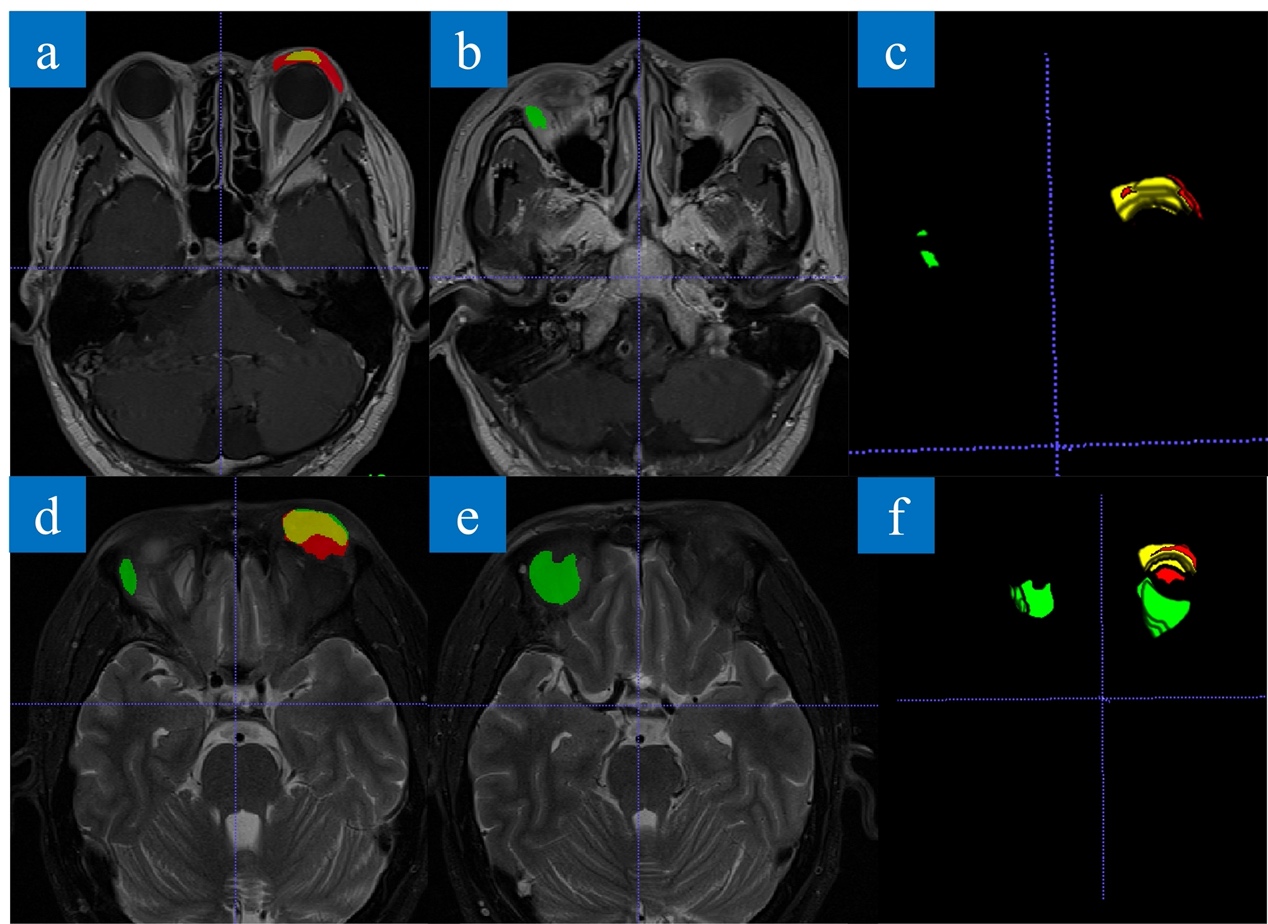

Supplement: Supplementary file 1 — Supplementary Material 1 [file 234_2024_3429_MOESM1_ESM.docx]
